# Healthy Dietary Interventions and Lipoprotein (a) Plasma Levels: Results from the Omni Heart Trial

**DOI:** 10.1371/journal.pone.0114859

**Published:** 2014-12-15

**Authors:** Bernhard Haring, Moritz C. Wyler von Ballmoos, Lawrence J. Appel, Frank M. Sacks

**Affiliations:** 1 Department of Internal Medicine I, Comprehensive Heart Failure Ctr, University of Würzburg, Bavaria, Germany; 2 Department of Cardiothoracic Surgery, Froedtert Memorial Hospital & Medical College of Wisconsin, Milwaukee, Wisconsin, United States of America; 3 Welch Center for Prevention, Epidemiology, and Clinical Research, Johns Hopkins University, Baltimore, Maryland, United States of America; 4 Department of Nutrition, Harvard School of Public Health, Boston, Massachusetts, United States of America; University of East Anglia, United Kingdom

## Abstract

**Background:**

Increased lipoprotein(a) [Lp(a)] levels are associated with atherosclerotic cardiovascular disease. Studies of dietary interventions on changes in Lp(a) are sparse. We aimed to compare the effects of three healthy dietary interventions differing in macronutrient content on Lp(a) concentration.

**Methods:**

Secondary analysis of a randomized, 3-period crossover feeding study including 155 (89 blacks; 66 whites) individuals. Participants were given DASH-type healthy diets rich in carbohydrates [Carb], in protein [Prot] or in unsaturated fat [Unsat Fat] for 6 weeks each. Plasma Lp(a) concentration was assessed at baseline and after each diet.

**Results:**

Compared to baseline, all interventional diets increased mean Lp(a) by 2 to 5 mg/dl. Unsat Fat increased Lp(a) less than Prot with a difference of 1.0 mg/dl (95% CI, −0.5, 2.5; p = 0.196) in whites and 3.7 mg/dl (95% CI, 2.4, 5.0; p<0.001) in blacks (p-value between races = 0.008); Unsat Fat increased Lp(a) less than Carb with a difference of −0.6 mg/dl, 95% CI, −2.1, 0.9; p = 0.441) in whites and −1.5 mg/dl (95% CI, −0.2, −2.8; p = 0.021) in blacks (p-value between races = 0.354). Prot increased Lp(a) more than Carb with a difference of 0.4 mg/dl (95% CI, −1.1, 1.9; p = 0.597) in whites and 2.2 mg/dl (95%CI, 0.9, 3.5; p = 0.001) in blacks (p-value between races = 0.082).

**Conclusion:**

Diets high in unsaturated fat increased Lp(a) levels less than diets rich in carbohydrate or protein with greater changes in blacks than whites. Our results suggest that substitutions with dietary mono- and polyunsaturated fatty acids in healthy diets may be preferable over protein or carbohydrates with regards to Lp(a).

**Trial Registration:**

Clinicaltrials.gov NCT00051350

## Introduction

Lipoprotein(a) [Lp(a)] is a plasma lipoprotein consisting of a cholesterol-rich LDL-like particle having one molecule of apolipoprotein B100 and an additional protein, apolipoprotein(a), attached to apoB via a disulfide bond [1], [2]. Epidemiologic studies have shown that elevated Lp(a) concentration is an independent risk factor for CVD attributed to its similarity to LDL and its structural homology of apolipoprotein(a) with plasminogen resulting in impaired thrombolysis [3], [4], [5], [6], [7]. Moreover, because Lp(a) spends a long time in circulation, i.e. 3–5 days, it has been argued that apo(a) impairs the removal of an atherogenic LDL-like particle from plasma which thereby increases its time in circulation for interactions with the vascular wall [8], [9].

Lp(a) concentration is regarded as largely genetically determined [10], [11], [12], [13]. Levels of Lp(a) are similar in men and women but vary among races with non-Hispanic whites having the lowest and blacks the highest [14]. Pharmacological therapies such as niacin and oral estrogens can lower Lp(a) level [9], [15], but investigations examining the effect of various dietary compositions on Lp(a) are sparse [16], [17]. So far, it has been reported that Lp(a) levels increase as a consequence of reducing and replacing dietary fat intake, and in particular saturated fat, with carbohydrates [16], [17]. However, to our knowledge no investigation has evaluated the influence of mono- or polyunsaturated fat compared to carbohydrates on Lp(a) levels in the context of a healthy dietary pattern. Moreover, data on the effect of dietary protein on Lp(a) are controversial [18], [19], [20], [21], [22].

In this report, we systematically compared the effects of three healthy diets differing in their macronutrient content on Lp(a) levels using a randomized cross-over design in an isocaloric nutritional setting. The primary outcome was the change in Lp(a) during a protein- or unsaturated fat-rich diet compared to a carbohydrate-rich diet. Because blacks have higher Lp(a) concentrations than whites, we investigated secondary racial differences in Lp(a) response to changing macronutrient compositions.

## Methods

### Study population

This is a secondary analysis of the ‘Optimal Macronutrient Intake Trial to Prevent Heart Disease’ (Omni Heart). The rationale and main results of the Omni Heart Trial have been published [23], [24]. In brief, 164 generally healthy participants were recruited from the population of the greater Baltimore, MD, and Boston, MA, areas. Participants were aged 30 years or older with blood pressure values above normal or stage 1 hypertension (systolic BP of 120–159 mm Hg or diastolic BP of 80–99 mm Hg). Participants with fasting LDL cholesterol exceeding 220 mg/dL, or triglycerides exceeding 750 mg/dL were not eligible for this trial. Race was self-identified via questionnaire. For this analysis, we only included black or white individuals. The Omni Heart trial aimed to recruit approximately 50% blacks as this group suffers from a high burden of hypertension and CVD. The study protocol was approved by the Institutional Review Boards at all affiliated institutions (Johns Hopkins University, Brigham & Women's Hospital, and the Harvard School of Public Health). Each participant provided written informed consent.

### Dietary Interventions

The Omni Heart Trial used a randomized, 3-period crossover design of controlled feeding to compare the effects of macronutrients on blood pressure and plasma lipids [23], [24]. Dietary interventions took place from April 2003 to June 2005. Three diets were tested – one diet emphasized carbohydrate [Carb], a second diet emphasized protein [Prot], and a third diet emphasized unsaturated fat [Unsat Fat]. A detailed description of these three diets is summarized in Table 1. All three diets were based on the ‘Dietary Approaches to Stop Hypertension’ (DASH) diet [20], [24], [25]. Each diet was reduced in saturated fat, cholesterol, and sodium, and rich in fruits, vegetables, fish, potassium, and other minerals at recommended levels. Protein mostly came from plants (legumes, grains, nuts, and seeds), but also included meat, poultry, egg product substitutes, dairy products and some soy products, although the amount was low (on average 7.3 g per day). The unsaturated fat diet emphasized mono- and polyunsaturated fat and included olive, canola, and safflower oils, as well as a variety of nuts and seeds, to meet its target fatty acid distributions. The type of carbohydrate as indicated by the total glycemic index was similar in all diets. All diets were held isocaloric.

**Table 1:** Baseline Intake and Nutrient Targets of Omni Heart Diets.

		Nutrient Targets of Omni Heart Diets		
	Baseline (± SD)	Carb	Prot	Unsat Fat
**Carbohydrate (% of energy)**	50±9	58	48	48
**Fat (% of energy)**	31±6	27	27	37
Saturated	11±3	6	6	6
Monounsaturated	13±3	13	13	21
Polyunsaturated	7±2	8	8	10
**Protein (% of energy)**	17±4	15	25	15
Meat	12±4	5.5	9	5.5
Plant and Dairy	5±2	9.5	16	9.5

After a 6-day run-in period, in which participants ate 2 days of meals from each study diet, participants were randomly assigned to 1 of 6 sequences of these feeding protocols [23], [24]. Each feeding period lasted 6 weeks. Throughout the feeding periods, participants were provided with all nutrition prepared in research kitchens. Meals were consumed on-site on weekdays. Weekend meals were consumed off-site. Participants were instructed to drink no more than 3 caffeinated beverage and no more than 2 alcoholic beverages per day. Trial participants were further asked not to change the level of exercise. Weight was measured each weekday and was kept stable by adjusting caloric levels, by adding 100-kcal cookies with the nutrient content of the assigned diet, or both. During controlled feeding, participants kept a diary recording compliance with study foods and consumption of non-study foods. According to these records, adherence was high i.e. all study food was consumed and no additional non-study food was eaten on 95 to 96% of person-days for each diet [24].

A washout period of 2 to 4 weeks separated the feeding periods. During the washout, participants ate their own food (self-selected diet) [23], [24]. The duration of washout periods (two to four weeks) in the Omni Heart Trial was determined following scientific and practical considerations [23]. As the biological half-life of Lp(a) is estimated to be approximately 3 to 4 days [8], a wash out period of 2 weeks for this secondary analysis of the Omni Heart Trial was considered sufficient.

The total study population of this secondary analysis was 155 individuals (Table 2). The sample size for each of the three comparisons was as follows Carb vs Unsat Fat: n = 151/Carb vs Prot: n = 151/Unsat Fat vs Prot: n = 150.

**Table 2:** Participant Characteristics at baseline^1^.

	All	Race		p-value
		Black	White	
**n**	155	89	66	-
**Female (%)**	46	56	32	-
**Age (y)**	54	52	56	0.046
	±11	±11	±10	
**BMI (kg/m2)**	30	31	29	0.016
	6	±6	±6	
**Homa Index**	2.4	2.6	2.3	0.506
	±2.3	±2.0	±2.8	
**Lp(a) (mg/dl)**	26.1	31.9	18.3	<0.001
	±23.0	±22.0	±22.2	
**Triglycerides (mg/dl)**	132.1	117.7	151.5	0.019
	±88.8	±88.0	±86.9	
**Total Chol (mg/dl)**	203.7	200.0	211.6	0.029
	±35.7	±32.6	±38.5	
**LDL-C (mg/dl)**	129.2	126.1	134.5	0.11
	±32.4	±31.4	±32.9	
**HDl-C (mg/dl)**	50.0	51.3	47.8	0.184
	±16.1	±17.9	±13.2	
**Apo B (mg/dl)**	84.2	80.0	90.1	0.018
	±26.2	±21.5	±30.9	
**Apo C-III (mg/dl)**	12.7	10.7	15.4	<0.001
	±8.0	±5.8	±9.7	
**Apo E (mg/dl)**	7.5	7.7	7.2	0.333
	±2.8	±2.9	±2.7	

1 HOMA, homeostasis model assessment; LDL-C, LDL cholesterol; HDL-C, HDL cholesterol; Apo, apolipoprotein. P values were derived from Student's t test comparing the different races. Values are unadjusted means ± SD.

### Laboratory measurements

Details of laboratory measurements have been published previously [23], [24], [26]. Briefly, blood samples of each participant were collected at baseline and after each of the three 6-week diet periods. Blood was drawn after a 12 h overnight fast into EDTA tubes. Plasma was separated by centrifugation at 1.500 rpm for 15 min at 4°C and immediately frozen at −70°C. The resulting serum was then shipped to the Core Laboratory for Clinical Studies (CLCS) of Washington University Medical School, St. Louis, Mo. Each participant's 4 samples were analyzed in the same batch in random order to reduce analytic variation. Batches consisted of samples of 5 or 9 participants, and analyses were completed within 5 days. All laboratory staff were blinded to the diet sequences of the study participants. Lp(a) was measured using an enhanced in-vitro diagnostic immunoturbidimetric assay for the quantitative determination of Lp(a) in serum (Wako Diagnostics: Lp(a) autokit). The assay utilized an anti-human Lp(a) antibody that does not cross-react with plasminogen or apoB and yields an insoluble aggregate that causes increased turbidity. The degree of turbidity was detected optically and was proportional to the amount of Lp(a) in the sample. Commercially available standards were used (Wako Chemicals, USA). All measurements were carried out at the Core Laboratory for Clinical Studies (CLCS) of Washington University Medical School, St. Louis, Mo.

### Statistics

The primary outcome for this secondary analysis of the Omni heart trial was change in Lp(a) during the Prot or Unsat diets compared to the Carb diet. Secondary, the effects of the intervention diets on Lp(a) in blacks compared to whites was a major aim. To assess changes from baseline and interdietary differences in Lp(a) in the overall study population as well as the effect modification by race we calculated mean differences (95% CI) using generalized estimating equations with an exchangeable correlation matrix accounting for study site and intervention order (Table 3). Similarly, generalized estimation equation models with interaction terms for a priori specified variables were calculated to estimate the respective effect sizes and effect modification (gender), as appropriate (S1 Table). Models without (Table 3 and S1 Table) and with (S2 Table and S3 Table) adjustment for baseline Lp(a) levels were calculated to evaluate the importance of baseline Lp(a) for the observed Lp(a) effect size after dietary intervention. Significance was defined as p<0.05 without adjustment for multiple comparisons. All analyses were conducted using STATA 11.0

**Table 3:** Lp(a) concentration (mg/dl) with diet by ethnicity: Changes from baseline and differences between diets reported as mean [95% CI].

		All			Whites			Blacks			
		Δ mean	[95%CI]	p-value	Δ mean	[95%CI]	p-value	Δ mean	[95%CI]	p-value	p-value between races
**Change from baseline**	**Carb**	3.2	(2.2, 4.2)	<0.001	2.2	(0.7, 3.7)	0.004	4.0	(2.7, 5.3)	<0.001	0.067
	**Unsat**	2.1	(1.1, 3.1)	<0.001	1.6	(0.1, 3.1)	0.038	2.5	(1.2, 3.8)	<0.001	0.372
	**Prot**	4.7	(3.7, 5.7)	<0.001	2.6	(1.1, 4.1)	0.001	6.2	(4.9, 7.5)	<0.001	<0.001
**Difference between study diets**	**[Carb] to [Unsat Fat]**	−1.1	(−0.1, −2.1)	0.026	−0.6	(−2.1, 0.9)	0.441	−1.5	(−0.2, −2.8)	0.021	0.354
	**[Carb] to [Prot]**	1.4	(0.4, 2.4)	0.005	0.4	(−1.1, 1.9)	0.597	2.2	(0.9, 3.5)	0.001	0.082
	**[Unsat Fat] to [Prot]**	2.5	(1.5, 3.5)	<0.001	1.0	(−0.5, 2.5)	0.196	3.7	(2.4, 5.0)	<0.001	0.008

## Results

### Participants

The characteristics of included participants at baseline examination are shown in total and by race in Table 2. Among 155 individuals in this analysis, 89 were black and 66 whites. Those belonging to the black racial group were more likely to be female, to be younger and have a higher BMI. Total apo C-III, apo B, cholesterol and triglyceride levels were significantly higher in whites compared to blacks. No difference in HOMA index was detected between races. The drop-out rate in the Omni Heart Trial was low for both races and distributed evenly across the 3 diets as reported previously [24], [26]. During the feeding program, mean body weight decreased on average from baseline by 1 kg, and this change occurred equally across all 3 diets in both blacks and whites [24], [26].

### Changes in Lp(a) concentration from baseline

**Figure 1:**
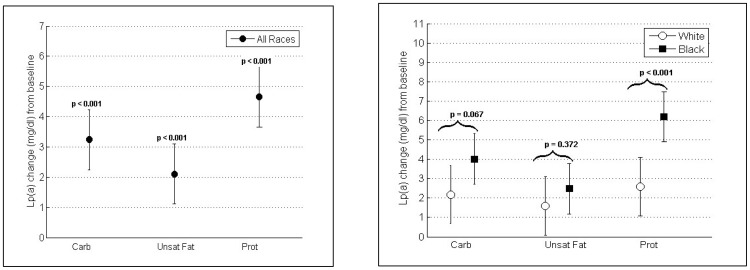
Mean [95% CI] changes in plasma Lp(a) from baseline by diet in all participants (Fig. 1a) and in blacks and whites (Fig. 1b) are shown. P-values for differences to baseline (Fig. 1a) and between races (Fig. 1b) are displayed. All interventions increased Lp(a). Between races, study diets increased Lp(a) in blacks more than in whites with significant differences after the Prot diet.

On self-selected diet at baseline mean Lp(a) was 26.1 mg/dl (95% CI, 22.5, 29.8) in the total study population, 18.3 mg/dl (95% CI, 12.9, 23.8) in whites and 31.9 mg/dl (95% CI, 27.3, 36.5) in blacks. Compared with baseline all dietary interventions increased mean Lp(a) levels in the total population and across whites and blacks (Table 3/Fig. 1). Largest Lp(a) increases were observed after the Prot diet (4.7 mg/dl; 95% CI, 3.7, 5.7; p<0.001) followed by the Carb (3.2 mg/dl; 95% CI, 2.2, 4.2; p<0.001) and Unsat Fat diet (2.1 mg/dl; 95% CI, 1.1, 3.1; p<0.001) in the total study population. After the Prot diet blacks responded with significantly larger Lp(a) increases from baseline than white participants (6.2 vs. 2.6 mg/dl; p-value between races <0.001). Otherwise Lp(a) changes from baseline between whites and blacks after a Carb (2.2 vs. 4.0 mg/dl; p-value between races = 0.067) or an Unsat Fat diet (1.6 vs. 2.5 mg/dl; p-value between races = 0.372) were not significantly different.

### Changes in Lp(a) concentration between interventional diets

**Figure 2:**
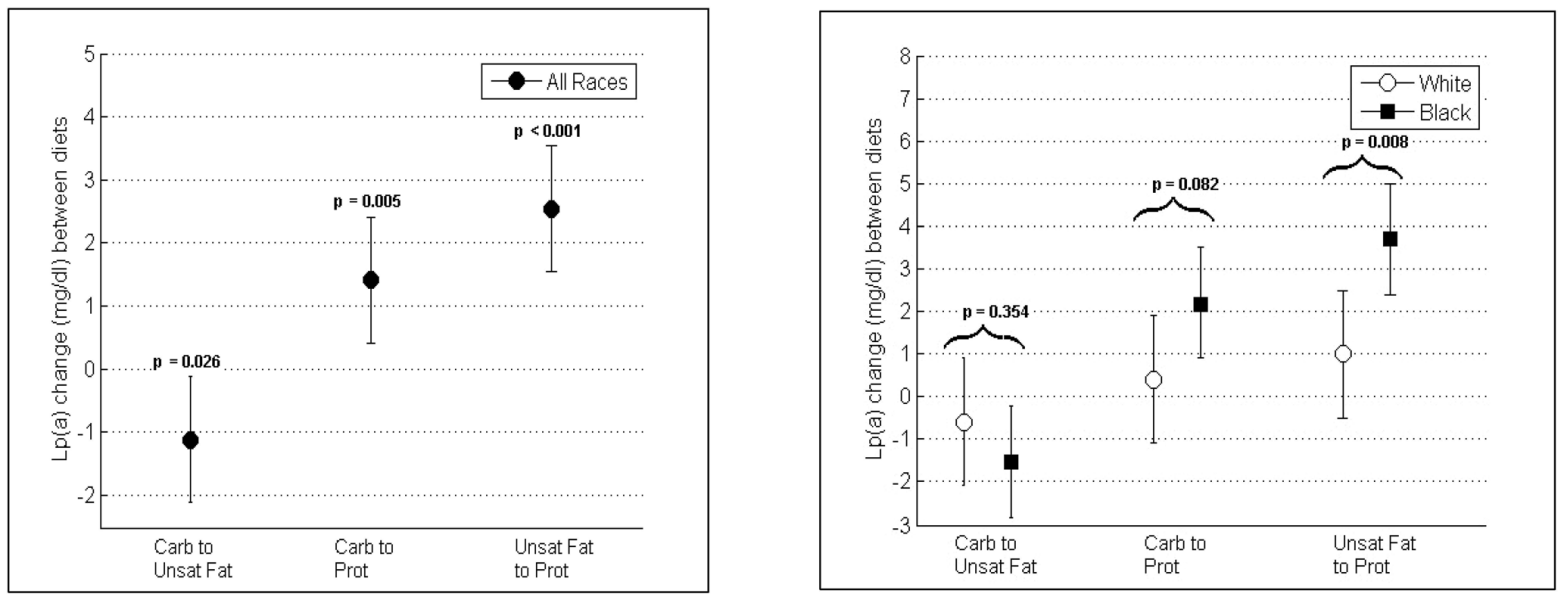
Mean [95% CI] changes in plasma Lp(a) between study diets in all participants (Fig. 2a) and in blacks and whites (Fig. 2b). P-values for differences between diets in all participants (Fig. 2a) and between blacks and whites (Fig. 2b) are displayed. Unsat Fat reduced Lp(a) compared to Prot or Carb. Blacks showed greater Lp(a) reductions when changing from Prot to Unsat Fat as compared to whites.

Compared with baseline the Unsat Fat diet increased mean Lp(a) levels less than the Carb diet (−1.1 mg/dl; 95% CI, −0.1, −2.1; p = 0.026) while the Prot diet increased Lp(a) concentration more than the Carb diet (1.4 mg/dl; 95% CI, 0.4, 2.4; p = 0.005) and more than the Unsat Fat diet (2.5 mg/dl; 95% CI, 1.5, 3.5; p<0.001). Among races Unsat Fat increased Lp(a) less than Carb with a difference of −0.6 mg/dl, 95% CI, −2.1, 0.9; p = 0.441) in whites and −1.5 mg/dl (95% CI, −0.2, −2.8; p = 0.021) in blacks (p-value between races = 0.354) (Table 3/Fig. 2).

Compared with baseline the Prot diet increased mean Lp(a) by 1.0 mg/dl (95% CI, −0.5, 2.5; p = 0.196) more than the Unsat Fat diet in whites and by 3.7 mg/dl (95% CI, 2.4, 5.0; p<0.001) in blacks (p-value between races = 0.008). Among races Prot increased Lp(a) more than Carb with a difference of 0.4 mg/dl (95% CI, −1.1, 1.9; p = 0.597) in whites and 2.2 mg/dl (95%CI, 0.9, 3.5; p = 0.001) in blacks (p-value between races = 0.082) (Table 3/Fig. 2).

We also examined gender differences in Lp(a) as a response to dietary changes (S1 Table). After consumption of a Carb diet female study participants exhibited significantly larger increases in Lp(a) compared to men (4.2 vs. 2.5 mg/dl; p = 0.087) but not after the Unsat Fat (2.4 vs. 1.9 mg/dl, p = 0.640) or the Prot (5.1 vs. 4.2 mg/dl; p = 0.370) diet. Overall, no significant effect modification by gender was observed (p = 0.368). Additional analyses on Lp(a) changes by race after dietary interventions and on effect modification by gender that adjusted for baseline Lp(a) did only marginally differ from levels without adjustment (S2 Table and S3 Table; S1 Figure and S2 Figure).

## Discussion

Healthy dietary interventions based on the DASH diet that emphasize carbohydrate, protein, or unsaturated fat have been shown to reduce atherogenic lipids and lipoproteins with diets rich in mono- or polyunsaturated fat or protein resulting in highest benefits [24]. Although Lp(a) is largely regarded as genetically determined [10], [12], [13], in our study DASH-type diets differing in macronutrient content increased Lp(a) compared to baseline. Among dietary interventions the Unsat Fat diet increased Lp(a) plasma levels less than the Carb and Prot diet. Blacks tended to respond with greater Lp(a) changes to dietary interventions compared to whites. Gender did not modify the effect of diet on Lp(a) concentrations.

The mechanisms by which macronutrient compositions influence Lp(a) levels are unknown. Previously, in the Delta study Ginsberg et al. reported increases of Lp(a) levels as total fat and in particular saturated fat intake were reduced and replaced by carbohydrates with intake of mono- and polyunsaturated fat or protein held constant [16]. Similarly, Shin et al. observed increases in Lp(a) after a high-fat low carbohydrate diet as compared to low-fat high carbohydrate study [17]. These data suggest that carbohydrate increases Lp(a) when it replaces either saturated or unsaturated fats [16], [17]. So far, limited evidence in humans is available comparing the effects on Lp(a) of saturated with unsaturated fats which indicate a slight increase in Lp(a) when replacing saturated fat with monounsaturated fat [27]. In monkeys, however, monounsaturated and polyunsaturated dietary fatty acids lowered Lp(a) concentrations compared to the saturated fatty acids, lauric and myristic acids [28], [29]. Specifically monkeys on a monounsaturated diet had decreased hepatic apo(a) messenger RNA abundance resulting in a decreased hepatic apo(a) transcription and decreased apo(a) production [28], [29]. On the other hand, Lp(a) has been observed to rise with increased intake of trans-fatty acids [16], [30], [31]. In the Omni Heart trial trans-fatty acids were avoided in all intervention diets as these are known to have deleterious effect on cardiovascular health [25], [26].

Among dietary factors potentially altering Lp(a), some studies found soy protein and isoflavones to increase plasma Lp(a) [20], [22] while others did not confirm this [18], [19], [21], [32]. The Prot diet consumed in our study contained little soy protein. The observed effect of protein on Lp(a) is therefore unlikely to be caused by soy and isoflavones but rather by other unknown reasons. Principally, dietary protein intake may affect apo(a) production rates as well as Lp(a) assembly rates, and catabolism rates. As plasma Lp(a) concentrations have been shown to be primarily controlled by the rate of synthesis rather than by the rate of clearance [8], [33], [34], dietary proteins may likely lead to an increase in Lp(a) production. Studies on the relationship of hormone treatment and drugs such as niacin with Lp(a) concentration support this notion [34]. Estrogen treatment of postmenopausal women reduces Lp(a) production and it thereby lowers plasma Lp(a) while it does not alter the catabolic rate of Lp(a) [9]. Similarly, niacin does not affect the catabolism of Lp(a) but decreases its synthetic rate and consequently lowers plasma Lp(a) levels [15]. Despite the rise in Lp(a) associated with the intake of a high protein compared to a high unsaturated fat DASH-type diet, given the beneficial effects of this dietary pattern on other plasma lipids such as LDL and on blood pressure, the healthy consequences may counteract and outweigh the potentially undesirable effects of an elevated Lp(a) concentration. Nonetheless, it is another aspect to consider when comparing Prot to Unsat Fat in the context of evaluating and analyzing the physiological changes of a healthy dietary pattern [24].

Blacks have higher average Lp(a) plasma concentrations than do whites [10], [12], [13]. Differences in dietary habits may explain only part of the racial difference in Lp(a). When consuming their usual diets at baseline, blacks reported consuming approximately 4% more calories from carbohydrate but 1 to 2% less from saturated or unsaturated fat than did whites [23], [24], [26]. Among ethnicities Lp(a) response to various dietary strategies is largely unknown [16]. Under controlled dietary conditions, our study shows that Lp(a) changes tended to be greater in blacks compared to whites across all diets compared to baseline self-selected diets, and among the three controlled diets. Our data therefore suggest that there is indeed some inherent racial difference in the metabolism of Lp(a) depending on macronutrient composition. We did not find gender differences in dietary Lp(a) response which is consistent with previous observations [16].

Limitations of this study include the relatively short duration of dietary periods as the effect of dietary interventions on Lp(a) could not be assessed in the long term. Furthermore, although participants were provided all of their food, we cannot exclude consumption of non-study food items as study adherence was monitored by self-report. Moreover, we are missing data on apo(a) isoforms and metabolism of Lp(a). Strengths are based on the crossover design, controlled feeding, and isocaloric dietary protocol of this study which allow parsimonious analysis with low risk of confounding. The risk of bias can be considered as low because of high rates of adherence to and high follow-up after all three diets.

In conclusion, interventional DASH-type diets differing in macronutrient content increased Lp(a) levels compared to baseline. Diets high in unsaturated fat increased Lp(a) less than diets rich in carbohydrate or protein with larger differences in blacks. Our results provide first systematic evidence for the changes of Lp(a) as a result of macronutrient composition and suggest that substitutions with dietary mono- and polyunsaturated fatty acids in healthy diets may be preferable over protein or carbohydrates with regards to Lp(a).

## Supporting Information

S1 Figure **Mean [95% CI] changes in plasma Lp(a) from baseline by diet after adjustment for baseline Lp(a) levels.** P-values for differences between races are displayed. Study diets increased Lp(a) in blacks more than in whites with significant differences after the Prot diet.
(TIFF)

S2 Figure **Mean [95% CI] changes in plasma Lp(a) between study diets after adjustment for baseline Lp(a).** P-values for differences between diets and between blacks and whites are displayed. Unsat Fat significantly reduced Lp(a) compared to Prot or Carb. Blacks showed greater Lp(a) reductions when changing from Prot to Unsat Fat as compared to whites.
(TIFF)

S1 Table **Effect modification by sex for changes in Lp(a) concentration (mg/dl): Changes from baseline and difference between diets reported as mean [95% CI].**
(DOCX)

S2 Table **Lp(a) concentration (mg/dl) with diet by ethnicity: Changes from baseline and difference between diets reported as mean [95%CI] after adjustment for baseline Lp(a) levels.**
(DOCX)

S3 Table **Effect modification by sex for changes in Lp(a) concentration (mg/dl): Changes from baseline and difference between diets reported as mean [95% CI] after baseline adjustment.**
(DOCX)

## References

[1] Frank S, Durovic S, Kostner GM (1996) The assembly of lipoprotein Lp(a). Eur J Clin Invest 26:109–114. 8904519 10.1046/j.1365-2362.1996.112255.x

[2] Kostner GM, Wo X, Frank S, Kostner K, Zimmermann R, et al (1997) Metabolism of Lp(a): assembly and excretion. Clin Genet 52:347–354. 9520125 10.1111/j.1399-0004.1997.tb04352.x

[3] Erqou S, Kaptoge S, Perry PL, Di Angelantonio E, Thompson A, et al (2009) Lipoprotein(a) concentration and the risk of coronary heart disease, stroke, and nonvascular mortality. JAMA 302:412–423. 19622820 10.1001/jama.2009.1063 PMC3272390

[4] Genser B, Dias KC, Siekmeier R, Stojakovic T, Grammer T, et al (2011) Lipoprotein (a) and risk of cardiovascular disease–a systematic review and meta analysis of prospective studies. Clin Lab 57:143–156. 21500721

[5] Nordestgaard BG, Chapman MJ, Ray K, Boren J, Andreotti F, et al (2010) Lipoprotein(a) as a cardiovascular risk factor: current status. Eur Heart J 31:2844–2853. 20965889 10.1093/eurheartj/ehq386 PMC3295201

[6] Ohira T, Schreiner PJ, Morrisett JD, Chambless LE, Rosamond WD, et al (2006) Lipoprotein(a) and incident ischemic stroke: the Atherosclerosis Risk in Communities (ARIC) study. Stroke 37:1407–1412. 16675734 10.1161/01.STR.0000222666.21482.b6

[7] Kronenberg F, Steinmetz A, Kostner GM, Dieplinger H (1996) Lipoprotein(a) in health and disease. Crit Rev Clin Lab Sci 33:495–543. 8989507 10.3109/10408369609080056

[8] Krempler F, Kostner GM, Bolzano K, Sandhofer F (1980) Turnover of lipoprotein (a) in man. J Clin Invest 65:1483–1490. 7410552 10.1172/JCI109813 PMC371487

[9] Su W, Campos H, Judge H, Walsh BW, Sacks FM (1998) Metabolism of Apo(a) and ApoB100 of lipoprotein(a) in women: effect of postmenopausal estrogen replacement. J Clin Endocrinol Metab 83:3267–3276. 9745440 10.1210/jcem.83.9.5116

[10] Boomsma DI, Kaptein A, Kempen HJ, Gevers Leuven JA, Princen HM (1993) Lipoprotein(a): relation to other risk factors and genetic heritability. Results from a Dutch parent-twin study. Atherosclerosis 99:23–33. 8461057 10.1016/0021-9150(93)90047-x

[11] Kamstrup PR, Benn M, Tybjaerg-Hansen A, Nordestgaard BG (2008) Extreme lipoprotein(a) levels and risk of myocardial infarction in the general population: the Copenhagen City Heart Study. Circulation 117:176–184. 18086931 10.1161/CIRCULATIONAHA.107.715698

[12] Kamstrup PR, Tybjaerg-Hansen A, Steffensen R, Nordestgaard BG (2009) Genetically elevated lipoprotein(a) and increased risk of myocardial infarction. JAMA 301:2331–2339. 19509380 10.1001/jama.2009.801

[13] Lanktree MB, Anand SS, Yusuf S, Hegele RA (2010) Comprehensive analysis of genomic variation in the LPA locus and its relationship to plasma lipoprotein(a) in South Asians, Chinese, and European Caucasians. Circ Cardiovasc Genet 3:39–46. 20160194 10.1161/CIRCGENETICS.109.907642

[14] Banerjee D, Wong EC, Shin J, Fortmann SP, Palaniappan L (2011) Racial and Ethnic Variation in Lipoprotein (a) Levels among Asian Indian and Chinese Patients. J Lipids 2011:291954. 21660301 10.1155/2011/291954 PMC3108091

[15] Seed M, O'Connor B, Perombelon N, O'Donnell M, Reaveley D, et al (1993) The effect of nicotinic acid and acipimox on lipoprotein(a) concentration and turnover. Atherosclerosis 101:61–68. 8216503 10.1016/0021-9150(93)90102-z

[16] Ginsberg HN, Kris-Etherton P, Dennis B, Elmer PJ, Ershow A, et al (1998) Effects of reducing dietary saturated fatty acids on plasma lipids and lipoproteins in healthy subjects: the DELTA Study, protocol 1. Arterioscler Thromb Vasc Biol 18:441–449. 9514413 10.1161/01.atv.18.3.441

[17] Shin MJ, Blanche PJ, Rawlings RS, Fernstrom HS, Krauss RM (2007) Increased plasma concentrations of lipoprotein(a) during a low-fat, high-carbohydrate diet are associated with increased plasma concentrations of apolipoprotein C-III bound to apolipoprotein B-containing lipoproteins. Am J Clin Nutr 85:1527–1532. 17556688 10.1093/ajcn/85.6.1527

[18] Jenkins DJ, Kendall CW, Jackson CJ, Connelly PW, Parker T, et al (2002) Effects of high- and low-isoflavone soyfoods on blood lipids, oxidized LDL, homocysteine, and blood pressure in hyperlipidemic men and women. Am J Clin Nutr 76:365–372. 12145008 10.1093/ajcn/76.2.365

[19] Meinertz H, Nilausen K, Hilden J (2002) Alcohol-extracted, but not intact, dietary soy protein lowers lipoprotein(a) markedly. Arterioscler Thromb Vasc Biol 22:312–316. 11834534 10.1161/hq0202.103998

[20] Nilausen K, Meinertz H (1999) Lipoprotein(a) and dietary proteins: casein lowers lipoprotein(a) concentrations as compared with soy protein. Am J Clin Nutr 69:419–425. 10075325 10.1093/ajcn/69.3.419

[21] Sacks FM, Lichtenstein A, Van Horn L, Harris W, Kris-Etherton P, et al (2006) Soy protein, isoflavones, and cardiovascular health: an American Heart Association Science Advisory for professionals from the Nutrition Committee. Circulation 113:1034–1044. 16418439 10.1161/CIRCULATIONAHA.106.171052

[22] Teede HJ, Dalais FS, Kotsopoulos D, Liang YL, Davis S, et al (2001) Dietary soy has both beneficial and potentially adverse cardiovascular effects: a placebo-controlled study in men and postmenopausal women. J Clin Endocrinol Metab 86:3053–3060. 11443167 10.1210/jcem.86.7.7645

[23] Carey VJ, Bishop L, Charleston J, Conlin P, Erlinger T, et al (2005) Rationale and design of the Optimal Macro-Nutrient Intake Heart Trial to Prevent Heart Disease (OMNI-Heart). Clin Trials 2:529–537. 16422313 10.1191/1740774505cn123oa

[24] Appel LJ, Sacks FM, Carey VJ, Obarzanek E, Swain JF, et al (2005) Effects of protein, monounsaturated fat, and carbohydrate intake on blood pressure and serum lipids: results of the OmniHeart randomized trial. JAMA 294:2455–2464. 16287956 10.1001/jama.294.19.2455

[25] Sacks FM, Svetkey LP, Vollmer WM, Appel LJ, Bray GA, et al (2001) Effects on blood pressure of reduced dietary sodium and the Dietary Approaches to Stop Hypertension (DASH) diet. DASH-Sodium Collaborative Research Group. N Engl J Med 344:3–10. 11136953 10.1056/NEJM200101043440101

[26] Furtado JD, Campos H, Sumner AE, Appel LJ, Carey VJ, et al (2010) Dietary interventions that lower lipoproteins containing apolipoprotein C-III are more effective in whites than in blacks: results of the OmniHeart trial. Am J Clin Nutr 92:714–722. 20826623 10.3945/ajcn.2009.28532 PMC2937582

[27] Berglund L, Lefevre M, Ginsberg HN, Kris-Etherton PM, Elmer PJ, et al (2007) Comparison of monounsaturated fat with carbohydrates as a replacement for saturated fat in subjects with a high metabolic risk profile: studies in the fasting and postprandial states. Am J Clin Nutr 86:1611–1620. 18065577 10.1093/ajcn/86.5.1611

[28] Brousseau ME, Ordovas JM, Nicolosi RJ, Schaefer EJ (1994) Effects of dietary fat saturation on plasma lipoprotein(a) and hepatic apolipoprotein(a) mRNA concentrations in cynomolgus monkeys. Atherosclerosis 106:109–118. 8018102 10.1016/0021-9150(94)90087-6

[29] Brousseau ME, Ordovas JM, Osada J, Fasulo J, Robins SJ, et al (1995) Dietary monounsaturated and polyunsaturated fatty acids are comparable in their effects on hepatic apolipoprotein mRNA abundance and liver lipid concentrations when substituted for saturated fatty acids in cynomolgus monkeys. J Nutr 125:425–436. 7876917 10.1093/jn/125.3.425

[30] Tholstrup T, Marckmann P, Vessby B, Sandstrom B (1995) Effect of fats high in individual saturated fatty acids on plasma lipoprotein[a] levels in young healthy men. J Lipid Res 36:1447–1452. 7595068

[31] Nestel P, Noakes M, Belling B, McArthur R, Clifton P, et al (1992) Plasma lipoprotein lipid and Lp[a] changes with substitution of elaidic acid for oleic acid in the diet. J Lipid Res 33:1029–1036. 1431582

[32] Crouse JR 3rd, Morgan T, Terry JG, Ellis J, Vitolins M, et al (1999) A randomized trial comparing the effect of casein with that of soy protein containing varying amounts of isoflavones on plasma concentrations of lipids and lipoproteins. Arch Intern Med 159:2070–2076. 10510993 10.1001/archinte.159.17.2070

[33] Rader DJ, Cain W, Zech LA, Usher D, Brewer HB Jr. (1993) Variation in lipoprotein(a) concentrations among individuals with the same apolipoprotein (a) isoform is determined by the rate of lipoprotein(a) production. J Clin Invest 91:443–447. 8432853 10.1172/JCI116221 PMC287951

[34] Kostner KM, Kostner GM (2004) Factors affecting plasma lipoprotein(a) levels: role of hormones and other nongenetic factors. Semin Vasc Med 4:211–214. 15478043 10.1055/s-2004-835380

