# Supplementary figures and images for: Healthy Dietary Interventions and Lipoprotein (a) Plasma Levels: Results from the Omni Heart Trial
Source: PLoS One. 2014 Dec 15;9(12):e114859. doi: 10.1371/journal.pone.0114859 (PMC4266632; doi:10.1371/journal.pone.0114859)

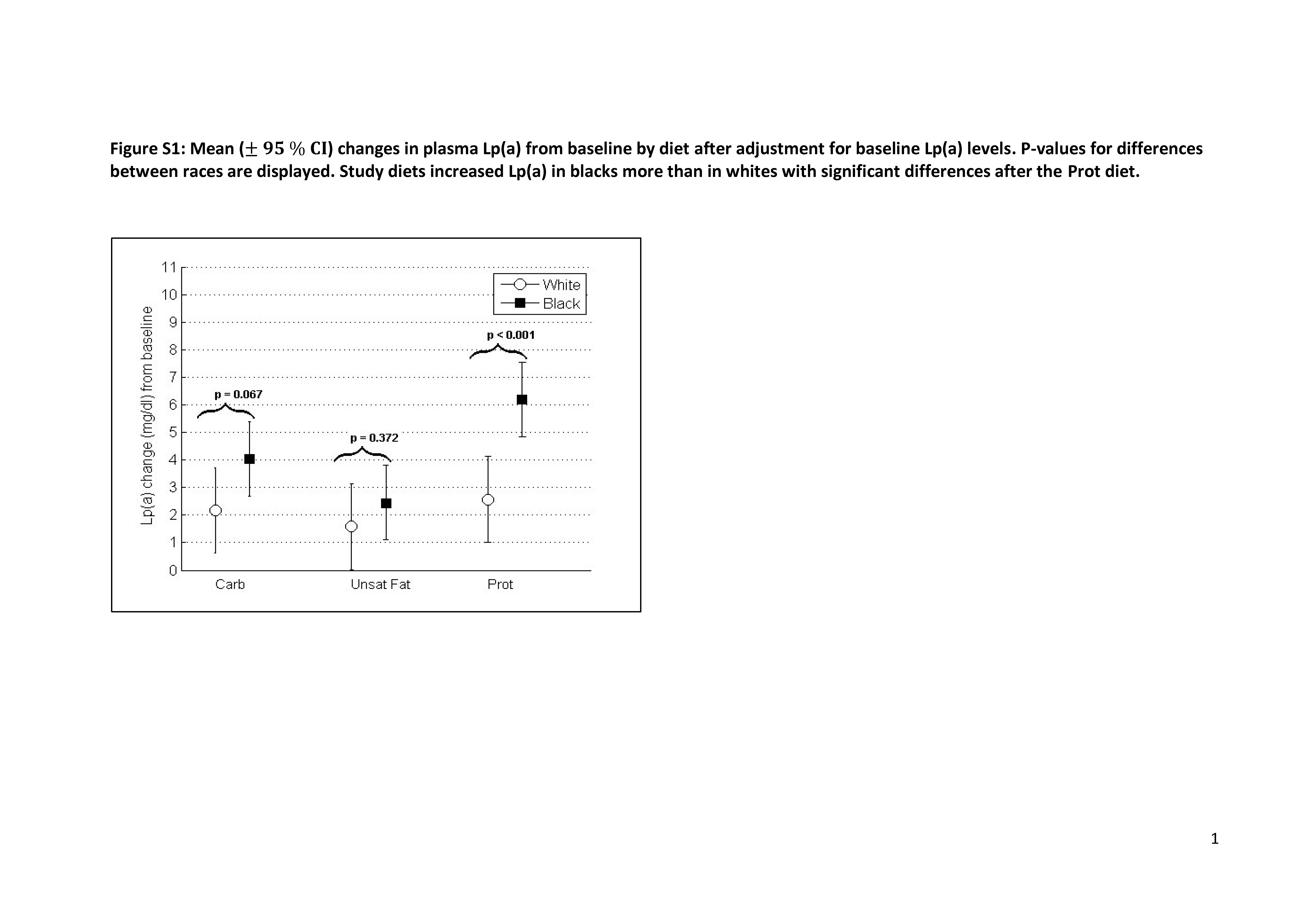

Supplement: S1 Figure — Mean [95% CI] changes in plasma Lp(a) from baseline by diet after adjustment for baseline Lp(a) levels. P-values for differences between races are displayed. Study diets increased Lp(a) in blacks more than in whites with significant differences after the Prot diet. (TIFF) [file pone.0114859.s001.tiff]

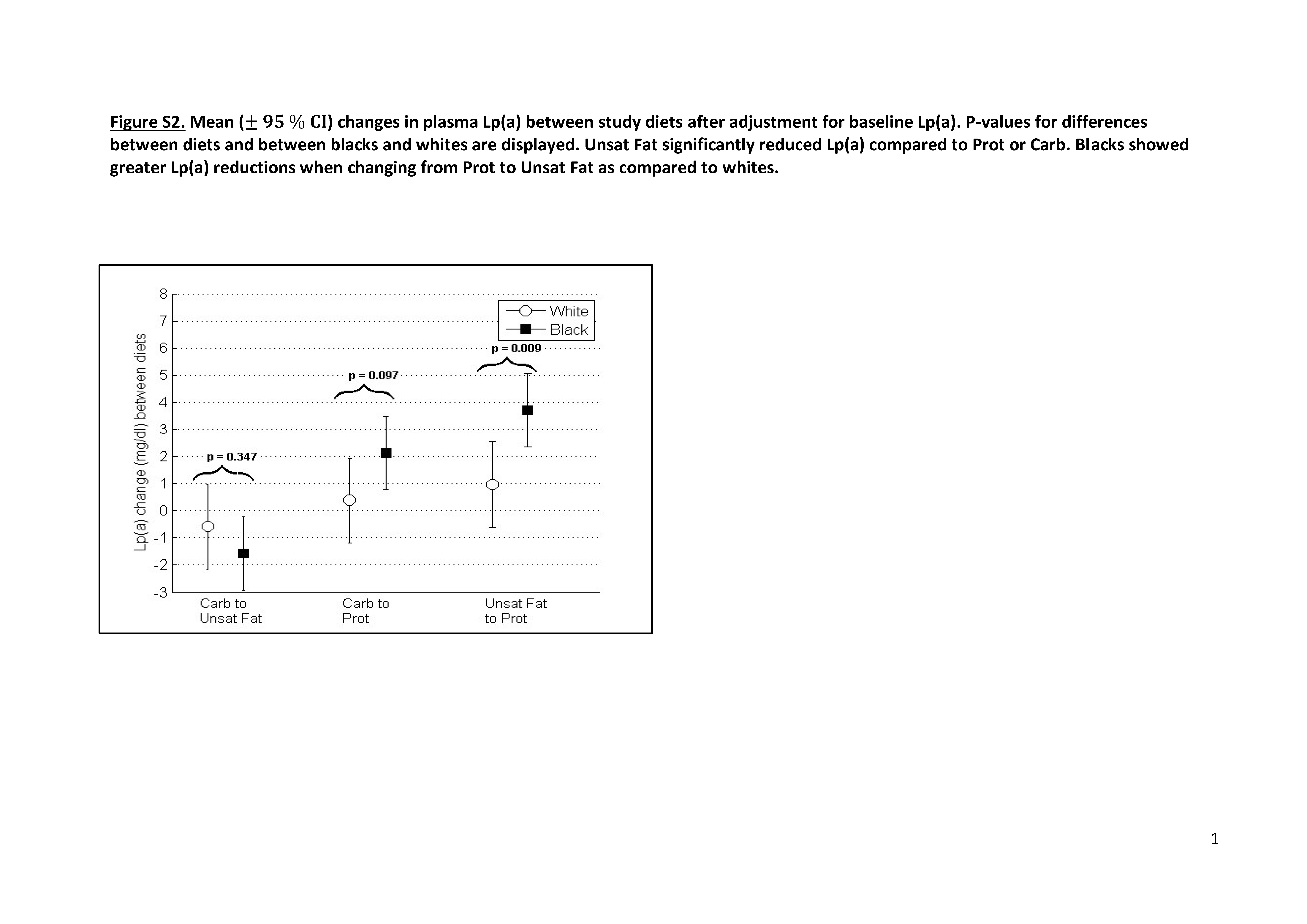

Supplement: S2 Figure — Mean [95% CI] changes in plasma Lp(a) between study diets after adjustment for baseline Lp(a). P-values for differences between diets and between blacks and whites are displayed. Unsat Fat significantly reduced Lp(a) compared to Prot or Carb. Blacks showed greater Lp(a) reductions when changing from Prot to Unsat Fat as compared to whites. (TIFF) [file pone.0114859.s002.tiff]
