# Supplementary material for: Healthy Dietary Interventions and Lipoprotein (a) Plasma Levels: Results from the Omni Heart Trial
Source: PLoS One. 2014 Dec 15;9(12):e114859. doi: 10.1371/journal.pone.0114859 (PMC4266632; doi:10.1371/journal.pone.0114859)
Supplement: S1 Table — Effect modification by sex for changes in Lp(a) concentration (mg/dl): Changes from baseline and difference between diets reported as mean [95% CI]. (DOCX) [file pone.0114859.s003.docx]

**Table S1:** **Effect modification by sex for changes in Lp(a) concentration (mg/dl): Changes from baseline and difference between diets reported as mean [95% CI]**

|  |  | **Male** | | **Female** | |  |
| --- | --- | --- | --- | --- | --- | --- |
|  |  | Δ mean | **[95%CI]** | Δ mean | **[95%CI]** | **p-value**  **(difference)** |
| **Change**  **from baseline** | **Carb** | 2.5 | (1.1, 3.8) | 4.2 | (2.7, 5.6) | 0.087 |
|  | **Unsat** | 1.9 | (0.5, 3.2) | 2.4 | (0.9, 3.8) | 0.640 |
|  | **Prot** | 4.2 | (2.9, 5.6) | 5.1 | (3.7, 6.0) | 0.370 |
|  |  |  |  |  |  |  |
| **Difference**  **between study diets** | **[Carb] to [Unsat Fat]** | -0.6 | (-1.9, 0.8) | -1.8 | (-0.3, -3.3) | 0.218 |
|  | **[Carb] to [Prot]** | 1.8 | (0.4, 3.1) | 1.0 | (-0.5, 2.4) | 0.421 |
|  | **[Unsat Fat] to [Prot]** | 2.3 | (1.0, 3.7) | 2.8 | (1.3, 4.3) | 0.670 |
| **Overall p-value for effect modification** | | | | | | 0.368 |
