# Supplementary material for: Healthy Dietary Interventions and Lipoprotein (a) Plasma Levels: Results from the Omni Heart Trial
Source: PLoS One. 2014 Dec 15;9(12):e114859. doi: 10.1371/journal.pone.0114859 (PMC4266632; doi:10.1371/journal.pone.0114859)
Supplement: S2 Table — Lp(a) concentration (mg/dl) with diet by ethnicity: Changes from baseline and difference between diets reported as mean [95%CI] after adjustment for baseline Lp(a) levels. (DOCX) [file pone.0114859.s004.docx]

**Table S2: Lp(a) concentration (mg/dl) with diet by ethnicity: Changes from baseline and difference between diets reported as mean [95%CI] after adjustment for baseline Lp(a) levels**

|  |  | **All** | | | **Whites** | | | **Blacks** | | |  |
| --- | --- | --- | --- | --- | --- | --- | --- | --- | --- | --- | --- |
|  |  | Δ mean | **[95%CI]** | **p-value** | Δ mean | **[95%CI]** | **p-value** | Δ mean | **[95%CI]** | **p-value** | **p-value between races** |
| **Change from baseline** | **Carb** | 3.3 | (2.2, 4.3) | <0.001 | 2.2 | (0.6, 3.7) | 0.006 | 4.1 | (2.7, 5.4) | <0.001 | 0.067 |
|  | **Unsat** | 2.1 | (1.1, 3.1) | <0.001 | 1.6 | (0.0, 3.2) | 0.045 | 2.4 | (1.1, 3.8) | <0.001 | 0.372 |
|  | **Prot** | 4.6 | (3.6, 5.7) | <0.001 | 2.6 | (1.0, 4.1) | 0.001 | 6.2 | (4.8, 7.5) | <0.001 | <0.001 |
|  |  |  |  |  |  |  |  |  |  |  |  |
| **Difference between study diets** | **[Carb] to**  **[Unsat Fat]** | -1.2 | (-0.1, -2.2) | 0.029 | -0.6 | (-2.1, 1.0) | 0.462 | -1.6 | (-0.2, -2.9) | 0.022 | 0.347 |
|  | **[Carb] to [Prot]** | 1.4 | (0.4, 2.4) | 0.009 | 0.4 | (-1.2, 2.0) | 0.625 | 2.1 | (0.8, 3.5) | 0.002 | 0.097 |
|  | **[Unsat Fat] to**  **[Prot]** | 2.5 | (1.5, 3.6) | <0.001 | 1.0 | (-0.6, 2.5) | 0.196 | 3.7 | (2.4, 5.1) | <0.001 | 0.009 |
