# Supplementary material for: Healthy Dietary Interventions and Lipoprotein (a) Plasma Levels: Results from the Omni Heart Trial
Source: PLoS One. 2014 Dec 15;9(12):e114859. doi: 10.1371/journal.pone.0114859 (PMC4266632; doi:10.1371/journal.pone.0114859)
Supplement: S3 Table — Effect modification by sex for changes in Lp(a) concentration (mg/dl): Changes from baseline and difference between diets reported as mean [95% CI] after baseline adjustment. (DOCX) [file pone.0114859.s005.docx]

**Table S3:** **Effect modification by sex for changes in Lp(a) concentration (mg/dl): Changes from baseline and difference between diets reported as mean [95%] after baseline adjustment**

|  |  | **Male** | | **Female** | |  | |
| --- | --- | --- | --- | --- | --- | --- | --- |
|  |  | Δ mean | **[95%CI]** | Δ mean | **[95%CI]** | **p-value**  **(difference)** |  |
| **Change from baseline** | **Carb** | 2.5 | (1.1, 3.8) | 4.2 | (2.7, 5.7) | 0.095 |  |
|  | **Unsat** | 1.9 | (0.5, 3.3) | 2.3 | (0.8, 3.9) | 0.674 |  |
|  | **Prot** | 4.2 | (2.8, 5.6) | 5.2 | (3.6, 6.7) | 0.372 |  |
|  |  |  |  |  |  |  |  |
| **Difference between study diets** | **[Carb] to [Unsat Fat]** | -0.6 | (-2.0, 0.8) | -1.9 | (-3.4, -0.3) | 0.214 |  |
|  | **[Carb] to [Prot]** | 0.9 | (0.4, 2.5) | 0.6 | (-0.5, 2.4) | 0.440 |  |
|  | **[Unsat Fat] to [Prot]** | 2.8 | (1.3, 3.3) | 2.8 | (1.3, 4.3) | 0.639 |  |
| **Overall p-value for effect modification** | | | | | | 0.380 |  |
